# Atmospheric Pressure Plasma-Treated Polyurethane Foam as Reusable Absorbent for Removal of Oils and Organic Solvents from Water

**DOI:** 10.3390/ma15227948

**Published:** 2022-11-10

**Authors:** Antonella Uricchio, Teresa Lasalandra, Eliana R. G. Tamborra, Gianvito Caputo, Rogério P. Mota, Fiorenza Fanelli

**Affiliations:** 1 Department of Chemistry, University of Bari “Aldo Moro”, Via Orabona 4, 70125 Bari, Italy; 2 Nanochemistry Department, Istituto Italiano di Tecnologia, Via Morego 30, 16163 Genoa, Italy; 3 Department of Physics, Faculty of Engineering and Science, São Paulo State University (UNESP), 12516-410 Guaratinguetá, SP, Brazil; 4 Institute of Nanotechnology (NANOTEC), National Research Council (CNR), c/o Department of Chemistry, University of Bari “Aldo Moro”, Via Orabona 4, 70125 Bari, Italy

**Keywords:** atmospheric pressure plasma, dielectric barrier discharge, surface engineering, plasma etching, nanotexturing, plasma deposition, polyurethane foam, oil absorbent, oil/water separation

## Abstract

This paper reports the optimization of a two-step atmospheric pressure plasma process to modify the surface properties of a polyurethane (PU) foam and, specifically, to prepare a superhydrophobic/superoleophilic absorbent for the removal of oils and nonpolar organic solvents from water. In particular, in the first step, an oxygen-containing dielectric barrier discharge (DBD) is used to induce the etching/nanotexturing of the foam surfaces; in the second step, an ethylene-containing DBD enables uniform overcoating with a low-surface-energy hydrocarbon polymer film. The combination of surface nanostructuring and low surface energy ultimately leads to simultaneous superhydrophobic and superoleophilic wetting properties. X-ray photoelectron spectroscopy, scanning electron microscopy and water contact angle measurements are used for the characterization of the samples. The plasma-treated PU foam selectively absorbs various kinds of hydrocarbon-based liquids (i.e., hydrocarbon solvents, mineral oils, motor oil, diesel and gasoline) up to 23 times its own weight, while it completely repels water. These absorption performances are maintained even after 50 absorption/desorption cycles and after immersion in hot water as well as acidic, basic and salt aqueous solutions. The plasma-treated foam can remove mineral oil while floating on the surface of mineral oil/water mixtures with a separation efficiency greater than 99%, which remains unaltered after 20 separation cycles.

## 1. Introduction

The increasing amount of industrial oily wastewaters, discharged for instance from petroleum refineries, thermoelectric power plants and mechanical industries, has become one of the most urgent environmental problems along with the frequent leakages of organic solvents due to various manufacturing processes [1,2]. Thus, the development of effective remediation strategies for the selective removal of oils and nonpolar organic solvents from water (commonly referred to as oil/water separation) has attracted global attention over the last few decades [1,3]. In particular, one of the most popular strategies toward this end involves the use of advanced functional materials with extremely opposite wetting behavior towards water and oils as well as nonpolar organic solvents [4]. In this context, both filtration and absorbent materials have been extensively studied [4, 5, 6, 7, 8, 9]. In particular, the use of absorbent materials with simultaneous superhydrophobic and superoleophilic properties (i.e., having a water contact angle greater than 150° and oil contact angle less than 10°) is considered very convenient [5,10]. For instance, open-cell foams (e.g., polymeric [11,12,13], carbon-based [14] and metal [15] sponges) have been regarded as promising candidates for the collection and removal of various kinds of oils and organic solvents from water, because they present highly porous three-dimensional (3D) structures, large pore volumes and thus high absorption capacities [8,12,16]. Among others, open-cell polyurethane (PU) foams are polymeric sponges widely preferred due to their availability, low cost [17,18,19,20], good chemical and thermal resistance, adequate flexibility, mechanical stability, light weight and low bulk density [18,21]. Interestingly, these latter properties [18,21] allow them to float on the water’s surface and selectively absorb oils and nonpolar organic solvents from it [22,23]. However, unmodified PU sponges show low oil/water separation capability because they are naturally hydrophilic [24] or slightly hydrophobic [22,25] and superoleophilic. Therefore, surface modifications are needed to achieve superhydrophobicity. The preparation of superhydrophobic PU foams requires changes in the surface chemical composition and morphology to respectively attain a low surface energy and a proper surface roughness at the micro- and/or nanoscale [12,16]. The surface functionalization either with a hydrophobic polymer coating [23,26] or a grafting agent [27] is typically used to reduce the surface energy. On the other hand, wet chemical etching [28] or nanoparticle anchoring [22,26,29] are commonly exploited to enhance surface roughness. To date, numerous methods have been applied, alone or in combination, to modify the surface properties of PU foams for oil/water separation. They include dip-coating [23,30], drop-casting [29], spray-coating [31], and various liquid phase and vapor phase methods [26,27,32]. However, it is still highly desirable to develop novel processing strategies to prepare polymer foams with a controlled surface chemistry, morphology and wettability. 

Over the last two decades, low-temperature atmospheric pressure (AP) plasmas have gained increasing interest as versatile tools for the surface engineering of many different materials [33,34,35]. Interestingly, recent studies have shown the remarkable potential of AP plasma processes for the effective surface modification of 3D porous materials [25,36,37,38,39,40,41,42,43] 

Up until now, few works have been dedicated to the use of AP plasmas for the preparation of functional materials to be used in oil/water separation [41,44,45,46,47]. Examples in the literature mainly focus on the surface processing of filtration materials, such as polymer or metal meshes, polymer membranes and cotton fabrics [45,46,47,48,49,50,51]. 

**Figure 1:**
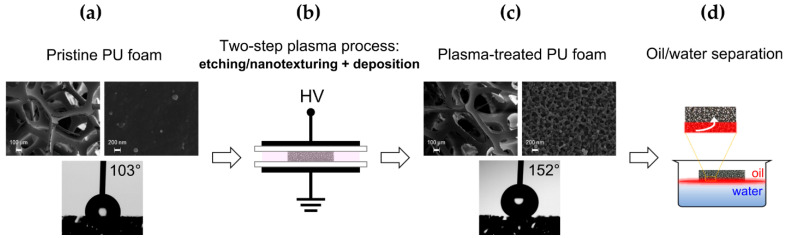
Overview of the present study in which an atmospheric pressure plasma process was optimized to prepare a superhydrophobic/superoleophilic PU foam able to selectively remove oils and nonpolar organic solvents from water: (**a**) representative SEM images of the pristine open-cell PU foam and photograph of a water droplet placed on it for WCA measurement; (**b**) surface modification of the foam by a two-step plasma process carried out using a parallel-plate DBD reactor and combining an etching/nanotexturing step and a subsequent deposition step; (**c**) representative SEM images of the plasma-treated PU foam and photograph of a water droplet placed on it for WCA measurement; (**d**) oil/water separation test carried out with the plasma-treated PU foam.

The aim of this work was the optimization of a low-temperature atmospheric pressure plasma process to modify commercial open-cell PU foams and obtain reusable absorbents for oil/water separation (Figure 1). The proposed two-step process combines plasma etching and thin-film deposition to achieve, respectively, surface nanotexturing and low surface energy, which ultimately lead to simultaneous superhydrophobic and superoleophilic wetting properties. Specifically, in the first step, an oxygen-containing atmospheric pressure dielectric barrier discharge (DBD) was employed to induce the texturing of the foam surfaces at the nanoscale; then, in the second step, an ethylene-containing DBD enabled uniform overcoating with a low-surface-energy hydrocarbon polymer film. Particular attention was devoted to evaluating the influence of the duration of each step in order to obtain superhydrophobic/superoleophylic foams for the efficient removal of oils and nonpolar organic solvents from water. 

## 2. Materials and Methods

### 2.1. Materials

A commercial open-cell foam (Angst+Pfister, Milan, Italy) characterized by polyester polyol-based polyurethane structure with pore density of 45 pores per inch and porosity of about 97% was used as substrate for the plasma treatments and absorption tests. 

Helium (Air Liquide, 99.999%, Milan, Italy), oxygen (Air Liquide, 99.999%, Milan, Italy) and ethylene (C_2_H_4_, Air Liquide, 99.95%, Milan, Italy) were used to feed the atmospheric pressure plasma. 

The liquids used for the absorption tests included bidistilled water, octane (Honeywell, purity = 99%, Seelze, Germany), toluene (Sigma-Aldrich, 99.9%, Steinheim, Germany), hexadecane (Sigma-Aldrich, 99%, Steinheim, Germany), light mineral oil (Sigma-Aldrich, viscosity at 40° = 14.2–17.2 cps, St. Louis, MO, USA), heavy mineral oil (Sigma-Aldrich, viscosity at 40° = 63.6–70.4 cps, St. Louis, MO, USA), motor oil (Petronas SYNTIUM MP 0W-30, viscosity at 40° of 54.1 cps, Turin, Italy), gasoline and diesel oil. HCl (Sigma-Aldrich, 37 wt%, Steinheim, Germany), NaOH (J.T.Baker, ≥95.5%, Deventer, Holland) and NaCl (Sigma-Aldrich, ≥99.5%, Buchs, Switzerland) were used to prepare acidic, basic and saline aqueous solutions, respectively. In oil/water separation tests, Sudan Red III (ABCR, Karlsruhe, Germany) was used to dye mineral oil for better visualization.

### 2.2. Atmospheric Pressure Plasma Processes

Plasma processes were performed using a home-made atmospheric pressure DBD reactor with parallel-plate electrode geometry (50 × 50 mm^2^ electrode size, 4 mm gas gap) [25] (Figure S1). The DBD was fed with He/O_2_ or He/C_2_H_4_ mixtures, and was generated by applying a sinusoidal AC high voltage (20.0 kHz, 1.3 kV_rms_). In particular, the plasma process used in this work consisted of two steps:

- Step 1—etching/nanotexturing, in which the plasma was fed with a He/O_2_ mixture at He flow rate of 6 standard liters per minute (slm) and O_2_ concentration of 0.5% (hereafter referred to as He/0.5% O_2_ mixture).
- Step 2—thin-film deposition, involving the use of a DBD fed with a He/C_2_H_4_ mixture at He flow rate and C_2_H_4_ concentration of 6 slm and 0.5%, respectively (hereafter referred to as He/0.5% C_2_H_4_ mixture).

The above-reported electrical and feed mixture conditions enabled the ignition of a filamentary DBD with average dissipated power of 12.0 ± 0.3 W (average specific power of 0.480 ± 0.012 W·cm^−2^). The discharge regime was dictated by the feed gas composition and was not affected by the presence of the porous samples. It was duly verified that discharge filaments’ formation did not cause damage to the foam struts and surfaces.

A lateral gas injection of the feed gas in the discharge region was used [36] (Figure S1). Gas canalization was accomplished by rectangular quartz bars placed along the electrode edges, parallel to the gas flow direction (Figure S1). Therefore, in the DBD system, the feed mixture flowed through a channel with rectangular cross-section of 50 × 4 mm^2^. During the plasma process, the foam samples were located at the center of the DBD region, sandwiched between the two dielectric-covered electrodes and thus in direct contact with them (Figure 1b and Figure S1a) [25,36,37,38]. In detail, as shown in Figure S1b, five foam samples (length × width × thickness = 20 × 10 × 4 mm^3^) were placed in the DBD system in order to form a rectangular strip (size 20 × 50 × 4 mm^3^) oriented perpendicularly with respect to the gas flow direction. In this way, the samples occupied the entire cross-section of the gas channel and, therefore, the feed gas was obliged to flow throughout their porous structure. It was preliminarily verified that the five samples treated by DBD in each process had comparable surface chemical composition, morphology and wettability. Two sets of experiments were carried out. In the first set, the duration of step 1 was varied from 0 to 15 min (etching/nanotexturing time, t_e/n_ = 0, 5, 10, 15 min), while the duration of step 2 (deposition time, t_d_) was kept fixed at 10 min. In the second set, the etching/nanotexturing time was kept constant at 10 min, while deposition times of 0, 5, 10 and 15 min were used.

### 2.3. Material Characterization

X-ray photoelectron spectroscopy (XPS) analyses were obtained with a PHI P5000 VersaProbe II scanning XPS microprobe spectrometer (ULVAC-PHI, Inc., Kanagawa, Japan), using a monochromatized Al Kα X-ray source (X-ray spot size = 100 μm, power = 14.8 W) as reported in [25,38]. The acquisition of wide scans (0–1400 eV) and high-resolution spectra (C 1s, O 1s, N 1s, Si 2p) was performed in fixed analyzer transmission mode, with a pass energy of 117.40 and 46.95 eV, respectively. The binding energy (BE) scale was corrected, taking the hydrocarbon component of the C 1s spectrum at 284.8 eV as reference. MultiPak software (Version 9.5.0.8, 30 October 2013, Ulvac-PHI, Inc., Kanagawa, Japan) was used for data processing [25,38]. XPS analyses were repeated on three plasma-treated samples (five spots per sample).

The morphological investigation was carried out by using a Zeiss SUPRA™ 40 field-emission scanning electron microscope (Carl Zeiss NTS GmbH, Oberkochen, Germany) [25,38]. The interior of the foams (i.e., cross-section) was observed after sample freeze-fracturing, as described in [36,38]. ImageJ software (1.53e, Wayne Rasband and contributors, National Institutes of Health, USA) was used to estimate from the scanning electron microscopy (SEM) images the thickness of the deposited coatings and the size of nanonodules formed on the foam surface (measurements on three plasma-treated samples). 

The wetting properties of the samples were evaluated with a KSV CAM200 optical contact angle meter (KSV Instruments, Helsinki, Finland). Static water contact angles (WCAs) were measured by depositing on the samples 5 μL distilled water droplets. Roll-off angles (ROAs) were determined by depositing 20 μL distilled water droplets on the sample fixed to a tiltable plate, and then inclining the plate slowly until the droplets started to move. Reported values are the average of measurements on three different samples (five measurements per sample). Water and light mineral oil absorption rates were determined indirectly by measuring with the KSV CAM200 instrument the time required for 5 μL liquid droplets to be completely absorbed by the porous samples.

### 2.4. Absorption and Oil–Water Separation Experiments

The absorption capacity (C) of the pristine and plasma-treated foams for water and various hydrocarbon-based liquids was evaluated from weight measurements according to the following procedure [26,32]. A dry pre-weighed foam sample was placed into a beaker containing the test liquid at room temperature and kept under vigorous shaking (120 rpm) for 60 s. Then, the sample was carefully taken out from the beaker and immediately weighed after draining off excess liquid. The absorption capacity of the foam, defined as the weight of liquid absorbed per unit weight of the foam at equilibrium, was determined using the following equation:

(1) $$C=\frac{m_{f}-m_{i}}{m_{i}}$$

where *m_i_* and *m_f_* are the weight of the sponge before and after liquid absorption, respectively. 

For each liquid, at least three samples were tested and average values were reported. It is worth specifying that, with all liquids used in this work, the foam samples reached absorption equilibrium after 15 s of immersion. The absorption capacity for water and light mineral oil was also verified after immersion of the foam for 60 min in hot water (at 50 °C and 80 °C), saturated NaCl aqueous solution (ca. 5 M), and acidic (pH = 2) and basic (pH = 10) aqueous solutions. 

The reusability tests were carried out by repeating the following absorption–desorption cycle procedure 50 times: (i) in the first step, the absorption capacity of the foam for water was determined after immersion for 60 s under vigorous shaking; (ii) then, the foam was squeezed with tweezers and dried at 30 °C for 60 min; (iii) in the third step, the absorption capacity of the foam for the selected organic liquid was determined after immersion for 60 s under vigorous shaking; (iv) finally, the foam was squeezed with tweezers several times, washed with ethanol and dried in an oven at 30 °C for 60 min [23,52,53]. 

In a typical oil/water separation test, a pre-weighed dry foam sample was placed into a beaker on the surface of a light mineral oil/water mixture and vigorously shaken for 5 min at room temperature. The mixture was prepared by adding a weighed amount of light mineral oil (ca. 0.200 g, corresponding to about the 45% of the amount of oil that the foam absorbs at equilibrium) to 40 mL of bidistilled water. After shaking, the sample was taken up and immediately weighed. The separation efficiency (*S*), defined as the ability of the foam to absorb the oil present in the mixture, was calculated according to Equation (2):

(2) $$S=\frac{m_{f}-m_{i}}{m_{i\;oil}}\times 100$$

where *m_i oil_* is the weight of light mineral oil present in the original mixture [28]. These tests were also carried out using mixtures of mineral oil with acidic (pH = 2), basic (pH = 12) or saturated NaCl solutions. 

## 3. Results

### 3.1. Plasma Process and Material Characterization

Figure 1 summarizes the present study, which focused on the surface modification of a commercial PU foam by atmospheric pressure dielectric barrier discharges to prepare a superhydrophobic/superoleophilic absorbent material to be able to selectively remove oils and nonpolar organic solvents from water. 

**Figure 2:**
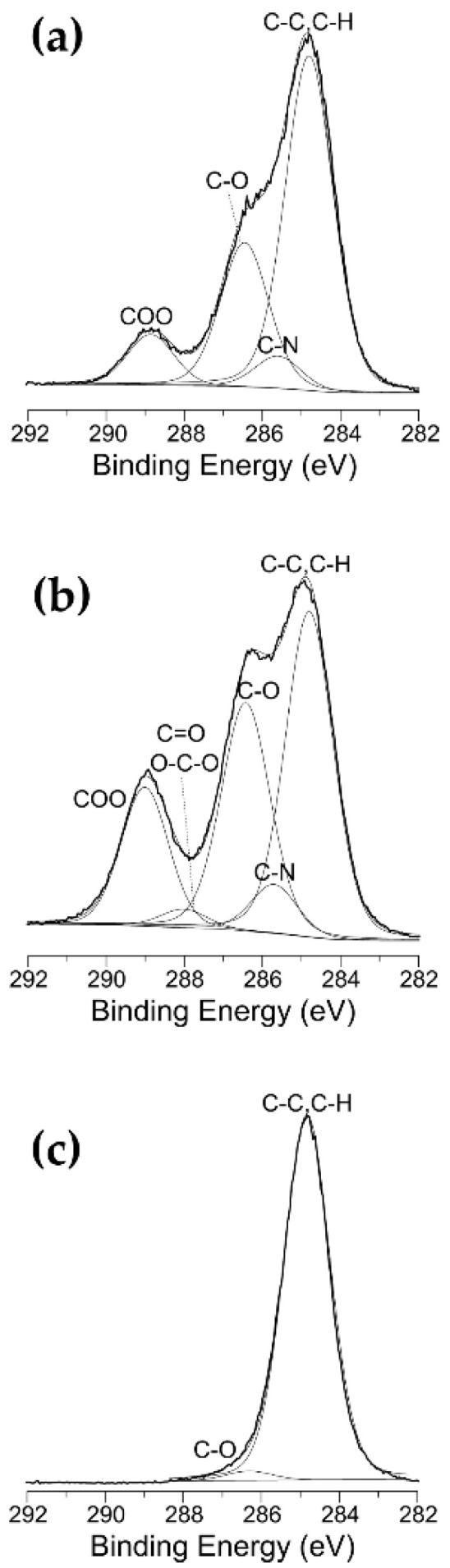
XPS C 1s spectra of (**a**) the pristine PU foam (reproduced with permission from 25), (**b**) the PU foam after step 1 (t_e/n_ = 10 min), and (**c**) the PU foam after the two-step plasma process (t_e/n_ = 10 min, t_d_ = 10 min).

The SEM images reported in Figure 1a evidence the porous structure of the commercial PU foam used in this work. It consists of a 3D continuous network of ligaments with quite smooth surfaces. XPS analyses of the pristine material revealed the presence of carbon, oxygen, nitrogen and silicon at surface atomic concentrations of 74.5, 20, 4.5 and 1%, respectively (Table 1). As reported in Table 2 and Figure 2a, the high-resolution C 1s XPS spectrum presents four components at 284.8 ± 0.2 eV (C-C, C-H, 60%), 285.6 ± 0.2 eV (C-N, 5.5%), 286.4 ± 0.2 eV (C-O, 26%), and 288.8 ± 0.2 eV (COO, 8.5%) [25,38]. 

The pristine foam exhibited a WCA of 103 ± 6° (Table 3) and absorbed light mineral oil quickly (a 5 μL oil droplet was absorbed at an absorption rate of ca. 10 μL·s^−1^), thus respectively showing inherent hydrophobicity and superoleophilicity. During the TOA measurements, the water droplets remained stuck on the sample and did not move even at a 90° inclination (i.e., pinned droplets). This suggested the need for surface modification to enhance hydrophobicity and, consequently, to achieve extremely opposite wetting behavior towards water and nonpolar liquids.

A two-step atmospheric pressure plasma process was therefore proposed and optimized. The process involved the sequential exposure of the polymer foam to a He/0.5% O_2_-fed DBD and a He/0.5% C_2_H_4_-fed DBD in order to obtain, respectively, surface nanotexturing and overcoating with a low-surface-energy hydrocarbon thin film. 

A detailed characterization of the samples was performed to examine the changes in surface chemistry, morphology and wettability that occur during each step of the process. It is worth noting that XPS and SEM investigations were carried out on both the exterior and interior surfaces of the plasma-treated samples, revealing a very similar surface composition and topography. This allowed us to confirm the uniformity of the surface modification over the entire porous sample, as already demonstrated in previous studies [25,36,38]. 

**Figure 3:**
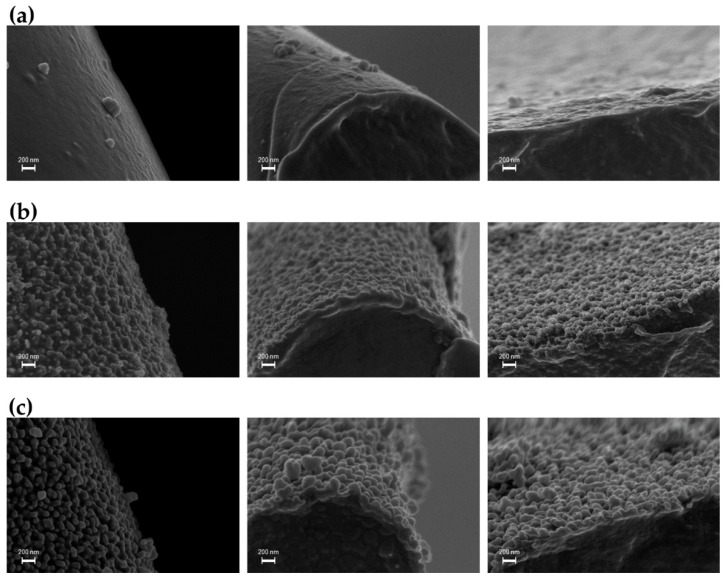
Representative SEM images of (**a**) the pristine PU foam, (**b**) the PU foam after step 1 (t_e/n_ = 10 min), and (**c**) the PU foam after the two-step plasma process (t_e/n_ = 10 min, t_d_ = 10 min). Images are taken in the sample interior (i.e., cross-section).

Table 1 shows that, as expected, the exposure of the PU foam to an O_2_-containing DBD in step 1 (t_e/n_ = 10 min) leads to a decrease in the XPS carbon atomic concentration and a concomitant increase in the oxygen atomic percentage with respect to the pristine sample; a certain increase in the nitrogen atomic percentage is also observed. In addition, the curve-fitting results of the high-resolution XPS C1s spectrum (Table 2, Figure 2b) indicate that the O_2_-containing plasma causes a remarkable decrease in the peak area percentage of the hydrocarbon C-C,C-H component (from 60% to 43%), a slight increase in the contributions of the components due to C-N and C-O moieties (as a whole, a 6.5% increase), the appearance of a new peak ascribed to the C=O and O-C-O functional groups (288.0 ± 0.2 eV, 2%) and, finally, a considerable increase in the component due to the carboxyl groups (from 8.5 to 17.5%) [25,54,55]. Overall, the XPS analyses clearly evidenced that the oxidation reactions take place at foam surfaces during step 1, as commonly reported in the literature on polymer treatments and etching with oxygen-containing plasmas [25,54,55]. These changes in surface chemical composition imparted a highly hydrophilic character to the foam, which after step 1 rapidly absorbed water at an absorption rate of ca. 25 μL·s^−1^ [25,56]. The SEM images in Figure 3b evidence that the He/O_2_ DBD treatment induces the texturing of the foam surfaces at the nanoscale. Similar modifications in polymer surface morphology have been widely reported so far. In fact, numerous studies have shown that polymer etching in oxygen-containing plasmas can lead to the formation of highly nanotextured surfaces [25,34,54,55,56,57,58]. 

In the second step of the plasma process, an ethylene-containing DBD was used to overcoat the nanotextured PU foam with a hydrocarbon thin film. The Fourier transform infrared (FTIR) spectrum of the deposited film in Figure S2 is characterized by the typical CH_2_ and CH_3_ stretching and bending signals (2800–3000 cm^−1^ and 1300–1600 cm^−1^, respectively) of a hydrocarbon polymer formed via the plasma polymerization of ethylene [59,60].

**Figure 4:**
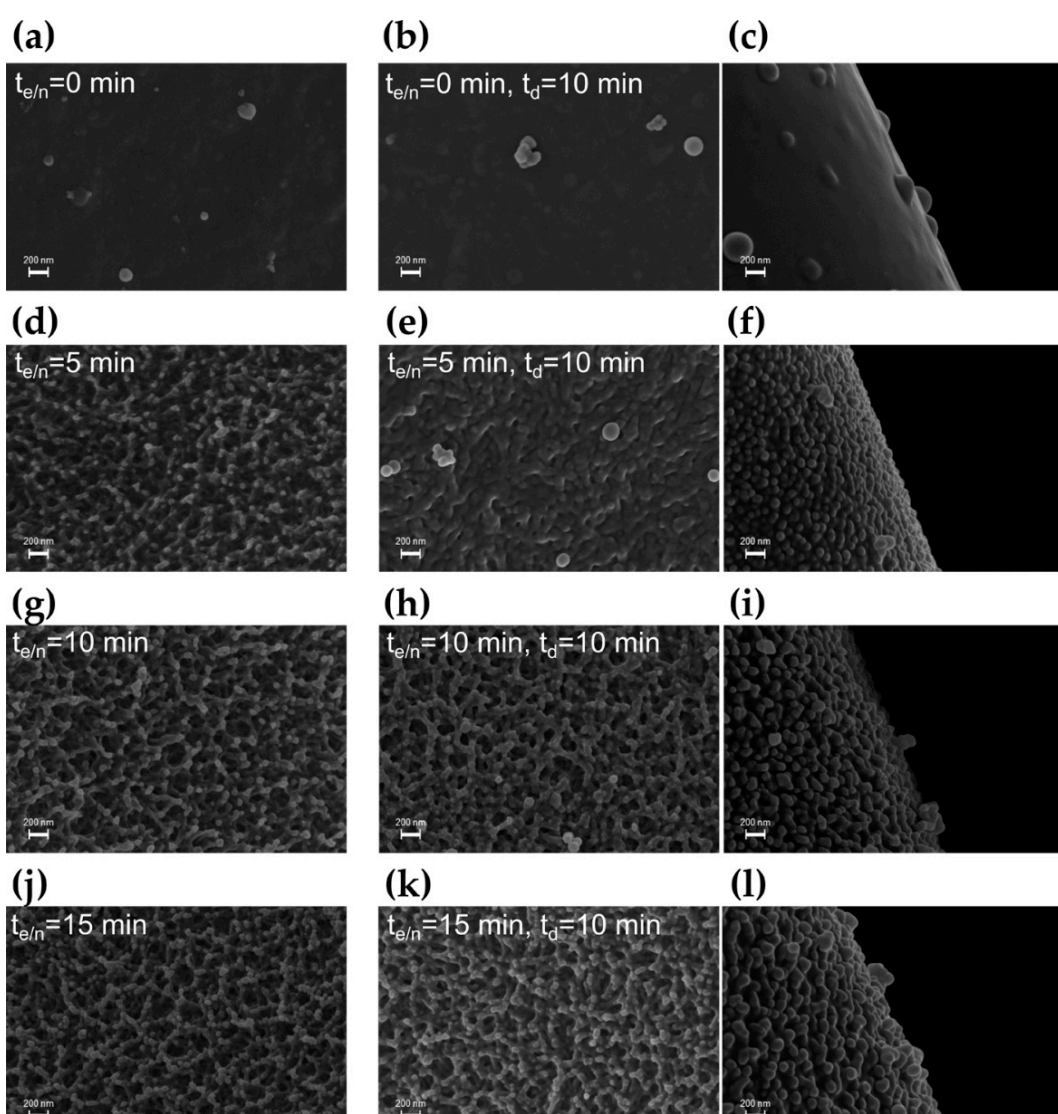
SEM images of the PU foams treated by the two-step plasma process, varying the duration of step 1 (t_e/n_ = 0, 5, 10, 15 min) and fixed the duration of step 2 fixed (t_d_ = 10 min): foam sample after step 1 with t_e/n_ = 0 min (**a**) and overcoating with a hydrocarbon polymer thin film in step 2 (**b**,**c**); foam sample after step 1 with t_e/n_ = 5 min (**d**) and overcoating in step 2 (**e**,**f**); foam sample after step 1 with t_e/n_ = 10 min (**g**) and overcoating in step 2 (**h**,**i**); foam sample after step 1 with t_e/n_ = 15 min (**j**) and overcoating in step 2 (**k**,**l**).

Interestingly, SEM observations of the pristine porous sample (not treated in step 1) plasma-coated using a He/0.5% C_2_H_4_-fed DBD showed that the hydrocarbon film presents quite a smooth morphology (Figure 4b,c and Figure S3, t_d_ = 10 min) and grows on the foam surfaces at an average rate of about 6 nm·min^−1^. The latter value was estimated from the SEM images of cross-sectioned ligaments, showing a coating thickness of 190 ± 40 nm after a 30 min deposition (Figure S4).

As reported in Table 1, the deposition of the hydrocarbon coating on the nanotextured foam in step 2 (t_d_ = 10 min) leads to a remarkable increase in the XPS atomic percentage of carbon (98%) and a concomitant decrease in the oxygen surface concentration (2%). In agreement, the XPS C 1s spectrum presents only two components, ascribed to the hydrocarbon (284.8 ± 0.2 eV, 98%) and C-O moieties (286.5 ± 0.2 eV, 2%) (Table 2, Figure 2c). On the other hand, the SEM images in Figure 3c show that, after the deposition of a thin hydrocarbon layer in step 2 (~60 nm thickness for t_d_ of 10 min), the nanoscale surface texture is preserved and apparently enhanced in the form’s nano-sized quasi-spherical features (i.e., nanonodules). It is important to note that, in spite of the substantial surface modifications induced by the two-step process, the plasma ignition into the foam interior does not affect its 3D porous structure, as can be appreciated from the low-magnification SEM image in Figure 1c. 

The coexistence of a low-surface-energy hydrocarbon polymer with a nanotextured topography enhanced the hydrophobicity of the foam, which after the two-step plasma process exhibited a WCA greater than 140° (Table 3), while maintaining superoleophilicity. It is worth specifying that the WCAs of a pristine (not nanotextured) foam and a glass slide after overcoating with the hydrocarbon polymer film were, respectively, 130 ± 5° and 100 ± 3°.

The effect of the duration of each step of the plasma process was therefore investigated with the aim of achieving superhydrophobic wetting properties. In the first set of experiments, the duration of the etching/nanotexturing step (t_e/n_) was varied in the range of 0–15 min, while the deposition time was kept fixed at 10 min in step 2. First of all, looking at the SEM images of the samples after step 1 (Figure 4a,d,g,j), it is possible to appreciate an increase in surface roughness as a function of t_e/n_. This trend seems to be maintained after the subsequent deposition step (Figure 4b,c,e,f,h,i,k,l). In particular, when t_e/n_ is increased from 5 to 15 min, the surface nanotexture obtained after the thin-film deposition becomes more pronounced as well, due to an enlargement of the nanonodules (Figure 4f,i,l). As estimated from SEM images, an increase in the average nodule size from about 90 to 150 nm can be observed. 

**Figure 5:**
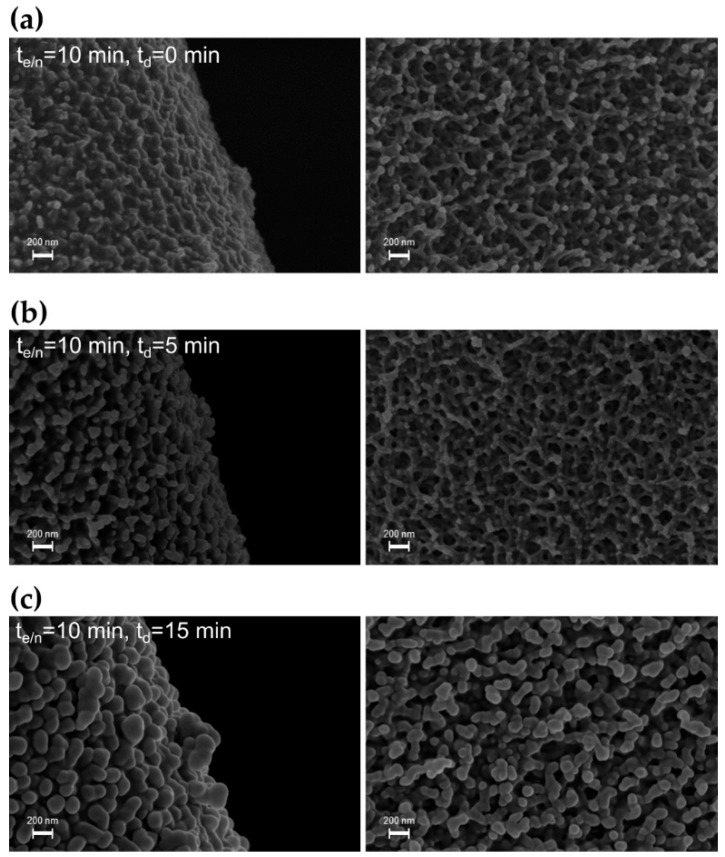
SEM images of the PU foams treated by the two-step plasma process, keeping fixed at 10 min the duration of step 1 and varying the duration of step 2: t_d_ of 0 min (**a**), 5 min (**b**), 15 min (**c**).

In a second set of experiments, the duration of the etching/nanotexturing step was kept constant at 10 min, while the duration of the deposition step was increased from 0 to 15 min. Figure 5 compares the SEM images of the foam after 10 min of etching/nanotexturing and subsequent deposition for 0, 5 and 15 min. It is possible to appreciate that the nodular protuberances on the foam surface become more evident and larger as a function of the duration of the deposition step. The nodule size is estimated to increase from approximately 100 nm to 220 nm with increasing the t_d_ from 5 to 15 min. 

It is important to note that, as assessed by XPS analyses, all foam samples presented the same surface chemical composition after the two-step plasma process, irrespective of the duration of each step. The surface chemistry of the plasma-treated foam remained dominated by the hydrocarbon film, featuring a very low O atomic concentration (Table 1 and Table 2, Figure 2c). 

Table 3 reports the WCA and roll-off angle (ROA) values of all plasma-treated foam samples prepared in this work. First of all, it can be observed that when the duration of both step 1 and step 2 ranged between 5 and 15 min, the WCA values were equal to or greater than 140°. The WCA was maximized after a two-step plasma process in which each step had a duration of 10 min. Under these processing conditions, a superhydrophobic wetting behavior (WCA = 152 ± 4°) was obtained. In addition, the ROA was also minimized (13 ± 2°), confirming the anti-wetting properties of the plasma-treated sample. In fact, the lower the ROA value, the more easily a water droplet is able to roll off the sample. The best-performing two-step plasma process, involving 10 min of etching/nanotexturing followed by 10 min of deposition, was therefore used for the surface modification of the foam samples to test the oil/water separation (Section 3.2).

To evaluate the durability of the surface modification after repeated compression, the superhydrophobic plasma-treated foam sample was submitted to 20 successive compression-release cycles using a pressure of 24.5 kPa, as described in the Supporting Material. Interestingly, no deterioration of the surface nanotexture and of the plasma-deposited hydrocarbon film was detected from SEM observations (Figure S5a). In contrast, when the hydrocarbon film was deposited directly on the pristine foam (no nanotexturing step), it appeared to be very prone to cracking and delamination upon repeated compression (Figure S5b). This evidence suggested the importance of the etching/nanotexturing step to attain mechanical interlocking and, thus, enhanced the stress resistance of the plasma-deposited coating [61,62]. In addition, it was also verified that the mechanical properties of the porous materials were almost not affected by the plasma process at all (Figure S6 and related description). Overall, it is noteworthy that the plasma process optimized in this work enables the uniform and effective modification of the surface properties of the porous material through independently controlling the two consecutive steps. This level of control represents a potential advantage and is rarely reported, for instance, in case of wet processes [11,23,28,29,30].

### 3.2. Oil–Water Separation

**Figure 6:**
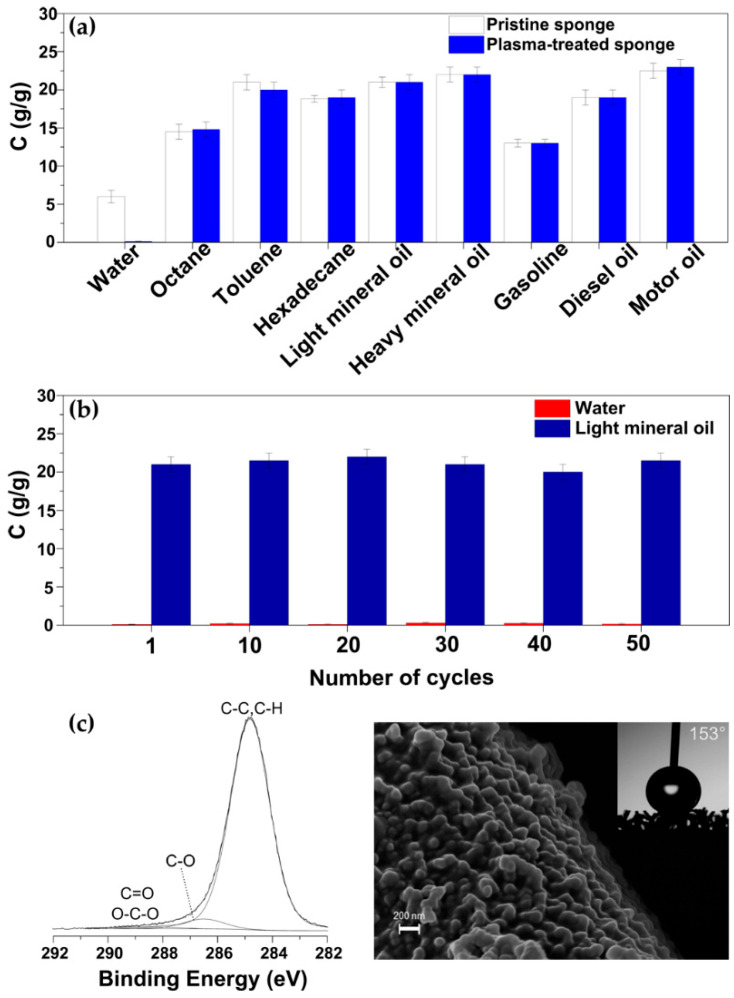
(**a**) Absorption capacity of the pristine and plasma-treated PU foams for water, various hydrocabon solvents and oils, gasoline, diesel oil and motor oil. The PU foam was modified by a two-step plasma process consisting of an etching/nanotexturing step (t_e/n_ = 10 min) followed by a thin-film deposition step (t_d_ = 10 min). (**b**) Absorption capacity of the plasma-treated foam for water and light mineral oil over 50 adsorption–desorption cycles. (**c**) Results from the characterization of the plasma-treated foam after 50 adsorption–desorption cycles using water and octane as test liquids: XPS C 1s signal, representative SEM image and photograph of a water droplet placed on the sample for WCA measurement.

Figure 6a shows the absorption capacity (C) of the pristine and superhydrophobic plasma-treated foams for water and various types of hydrocarbon-based liquids. The latter includes various hydrocarbon solvents, light and heavy mineral oil, gasoline, diesel oil and motor oil. 

First of all, it can be observed that both samples effectively absorb the hydrocarbon-based liquids up to 23 times their own weight. C seems to vary for the different hydrocarbon solvents and oils (15–23 g/g), mainly depending on their density and viscosity, as already reported in the literature [23,32]. On the other hand, for each tested organic liquid, the absorption capacity of the foam does not change appreciably after the two-step plasma process. In contrast, the plasma process leads to a drastic reduction in the water absorption capacity. The superhydrophobic plasma-treated foam exhibits a C value of approximately 0.1 g/g, considerably lower than that of the pristine sample (~6.0 g/g). The fact that the pristine foam had a slightly hydrophobic behavior (Figure 1a, Table 3) explains why it was unable to effectively repel water during the absorption experiments and confirms the necessity of optimizing a surface modification method. This further confirms the importance of the two-step plasma process in order to achieve superhydrophobicity and a consequent further reduction in water absorption. Interestingly, it was found that the absorption properties of the superhydrophobic plasma-treated foam for water and light mineral oil remained unchanged even after 60 min of immersion in acidic, basic, and saturated salt solutions and in hot water (Figure S7). 

The reusability of the plasma-treated foam was also investigated for all liquids used in this work, enlightening the excellent stability of the absorption performances after up to 50 absorption-desorption cycles. For instance, Figure 6b shows no noticeable changes in the C values for both water and light mineral oil over 50 absorption–desorption cycles, likely due to the durability of the surface modification. In-depth material characterizations were therefore performed to confirm the stability of the surface chemical composition, morphology and wettability of the plasma-treated foam after 50 octane absorption–desorption cycles (Figure 6c). XPS analyses revealed the presence of carbon and oxygen at atomic concentrations of 95 and 5%, respectively, comparable to those of the as-treated sample (Table 1). Accordingly, the high-resolution XPS C 1s spectrum (Figure 6c) remained dominated by the hydrocarbon component and showed very low contributions ascribed to oxygen-containing functionalities (C-O, 4% and O-C-O, C=O, 1%). Moreover, as shown in Figure 6c, after 50 absorption–desorption cycles, the plasma-treated sample still presented the distinctive surface nanotexture and superhydrophobic behavior (WCA > 150°). The recyclability performance of the plasma-treated foam seems high as compared to previous works on the surface modification of PU foams for oil/water separation [21,22,23,26,27,28,29,30]. In addition, it is noteworthy that previous studies reporting the recyclability of absorbent materials for oil/water separation are very rarely accompanied by a detailed material characterization after their use in applicative tests.

**Figure 7:**
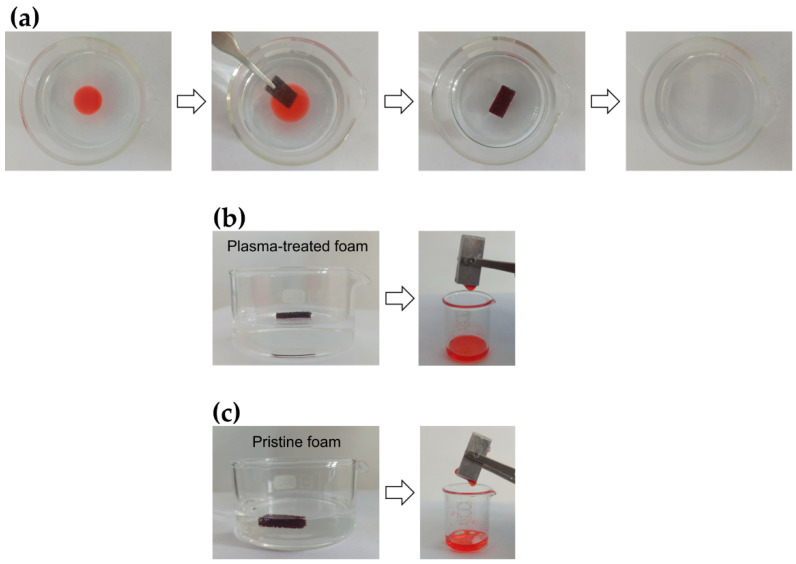
(**a**) Photographs of a typical oil/water separation experiment carried out using a PU foam sample modified by the two-step plasma process optimized in this work (t_e/n_ = 10 min, t_d_ = 10). During the separation experiment, the foam floats on the surface of a light mineral oil/water mixture. The transparent liquid is water, while the red liquid is light mineral oil dyed with Sudan Red III for better visualization. Photographs of the foam samples floating in a beaker containing the light mineral oil/water mixture and of the liquid collected by sample squeezing: (**b**) plasma-treated foam, (**c**) pristine foam.

Oil–water separation experiments were carried out with the pristine and plasma-treated foams to evaluate their ability to remove low-density oils floating on the water’s surface (Figure 7a). Figure 7b shows that when the superhydrophobic plasma-treated sponge was placed in a beaker containing a mixture of bidistilled water (i.e., the transparent higher-density liquid) and light mineral oil (i.e., the red-dyed lower-density liquid), it was able to float on the mixture’s surface and selectively absorb only the oil. This can be appreciated from the photograph taken during mechanical squeezing, which shows that only the mineral oil (i.e., the red-dyed liquid) was collected in the small beaker when the plasma-treated sample was squeezed. The plasma-treated foam exhibited a high separation efficiency (>99%), which remained unaltered after 20 separation cycles. Interestingly, Figure S8 shows that the *S* value of the plasma-treated foam was also greater than 99% when the separation tests were carried out using mixtures of mineral oil with acidic (pH = 2), basic (pH = 12) or saturated NaCl aqueous solutions as well as when heating the mineral oil/water mixture at 50 °C and 80 °C. In contrast, Figure 7c clearly shows that the pristine foam sinks below the surface of the mineral oil/water mixture during the separation experiment. This happens because the foam absorbs not only the oil floating on the water’s surface, but also a certain amount of water (i.e., the denser liquid), as confirmed by the photograph showing that both water and mineral oil were recovered by sample squeezing (Figure 7c). In agreement with these observations, weight measurements confirmed that the untreated sponge absorbs a liquid amount that is 25% greater than the amount of oil present in the original mixture (i.e., the *S* value of the pristine foam is 125%, Equation (2)).

## 4. Conclusions

In this study, an atmospheric pressure plasma process was developed to modify the surface properties of a commercial PU foam and, specifically, prepare a superhydrophobic/superoleophilic sorbent for the removal of oils and nonpolar organic solvents from water. The process involved the etching/nanotexturing of the foam surfaces in an O_2_-containing DBD, followed by overcoating with a low-surface-energy hydrocarbon film deposited in a C_2_H_4_-containing DBD. Our results illustrated the effective modification of the surface chemical composition and topography of the polymer foam. The plasma-treated foam showed a high absorption capacity for various types of hydrocarbon-based liquids, while completely repelling water. Furthermore, it exhibited remarkable reusability and durability after repeated adsorption–desorption cycles and immersion in corrosive aqueous solutions and hot water. The plasma-treated foam was able to remove mineral oil while floating on the surface of mineral oil/water mixtures. Overall, the results obtained in this study confirmed dielectric barrier discharges at atmospheric pressure are powerful tools for the effective and uniform surface modification of porous materials.

## Figures and Tables

**Table 1:** XPS surface atomic concentrations of the pristine PU foam, the PU foam after step 1, and the PU foam after the two-step plasma process. Step 1—etching/nanotexturing: He/0.5% O_2_ fed DBD, 20 kHz, 1.3 kV_rms_, 10 min; step 2—thin-film deposition: He/0.5% C_2_H_4_ fed DBD, 20 kHz, 1.3 kV_rms_, 10 min.

Sample	C at %	O at %	N at %	Si at %
Pristine foam	74.5 ± 4.0	20.0 ± 1.0	4.5 ± 0.2	1.00 ± 0.10
Plasma-treated foam/Step 1	64 ± 3	28.0 ± 1.5	7.0 ± 0.3	1.00 ± 0.10
Plasma-treated foam/Step 1+2	98.0 ± 1.0	2.0 ± 1.0	--	--

**Table 2:** Curve fitting results of C 1s XPS spectra of the pristine PU foam, the foam after step 1 (t_e/n_ = 10 min), and the foam after the two-step plasma process (t_e/n_ = 10 min, t_d_ = 10 min).

Sample	C-C, C-H Peak Area %	C-N Peak Area %	C-O Peak Area %	C=O, O-C-O Peak Area %	COO Peak Area %
Pristine foam	60 ± 4	5.5 ± 0.5	26 ± 2	--	8.5 ± 0.5
Plasma-treated foam/Step 1	43 ± 3	6.5 ± 0.5	31 ± 2	2.0 ± 0.2	17.5 ± 1.0
Plasma-treated foam/Step 1+2	98.0 ± 1.0	--	2.0 ± 1.0	--	--

**Table 3:** Water contact angle (WCA) and roll-off angle (ROA) of the PU foam before and after the two-step plasma process with different durations of each step (0, 5, 10, 15 min).

Sample	Step 1 Duration, t_e/n_ (min)	Step 2 Duration, t_d_ (min)	WCA (°)	ROA (°)
Pristine foam	-	-	103 ± 6	pinned
Plasma-treated foam	0	10	130 ± 5	pinned
	5	10	140 ± 3	47 ± 5
	10	10	152 ± 4	13 ± 2
	15	10	148 ± 3	32 ± 5
Plasma-treated foam	10	0	absorption	-
	10	5	145 ± 3	45 ± 4
	10	10	152 ± 4	13 ± 2
	10	15	141 ± 3	35 ± 4

## Data Availability

The data in this study are available from the corresponding author upon request.

## Supplementary Materials

The following supporting information can be downloaded at: https://www.mdpi.com/article/10.3390/ma15227948/s1. Figure S1: schematic of the atmospheric pressure DBD reactor and photograph showing the sample positioning in the DBD cell; Figure S2: FTIR spectrum of the thin film deposited by He/0.5% fed C_2_H_4_ fed DBD; Figure S3: SEM images of a PU foam sample coated with a hydrocarbon polymer thin film deposited (t_d_ = 10 min) and photograph of a water droplet deposited on the sample; Figure S4: SEM images of a PU foam sample coated with a hydrocarbon polymer thin film deposited by a He/0.5% C_2_H_4_ fed DBD for 30 min; Figure S5: SEM images of plasma-treated PU foam samples after 20 compression-release cycles; Figure S6: Young’s modulus (a) and tensile strength (b) of the pristine and plasma-treated PU foams; Figure S7: absorption capacity for water and light mineral oil of the superhydrophobic/superoleophilic PU foam after 60 min immersion in acidic, basic and saturated NaCl aqueous solutions as well as in hot water; Figure S8: separation efficiency of the superhydrophobic/superoleophilic PU for mixtures of light mineral oil with bidistilled water, acidic, basic and saturated NaCl aqueous solutions.
