# Supplementary material for: Atmospheric Pressure Plasma-Treated Polyurethane Foam as Reusable Absorbent for Removal of Oils and Organic Solvents from Water
Source: Materials (Basel). 2022 Nov 10;15(22):7948. doi: 10.3390/ma15227948 (PMC9693071; doi:10.3390/ma15227948)
Supplement: Supplementary file 1 [file materials-15-07948-s001.zip › materials-1988706-supplementary.pdf]

materials

Supplementary Material

Atmospheric pressure plasma-treated polyurethane foam as reusable absorbent for removal of oils and organic solvents from water

Antonella Uricchio [1,†], Teresa Lasalandra [1,†], Eliana R. G. Tamborra [1], Gianvito Caputo [2], Rogério P. Mota [3] and Fiorenza Fanelli [4,*]

[1] Department of Chemistry, University of Bari "Aldo Moro", via Orabona 4, 70125 Bari, Italy
[2] Nanochemistry Department, Istituto Italiano di Tecnologia, via Morego 30, 16163 Genoa, Italy
[3] Department of Physics, Faculty of Engineering and Science, São Paulo State University (UNESP), 12516-410 Guaratinguetá, SP, Brazil
[4] Institute of Nanotechnology (NANOTEC), National Research Council (CNR), c/o Department of Chemistry, University of Bari "Aldo Moro", via Orabona 4, 70125 Bari, Italy
* Correspondence: fiorenza.fanelli@cnr.it; Tel.:+39-080-5442227 (F.F.)
† These authors contributed equally to this work.

(a)

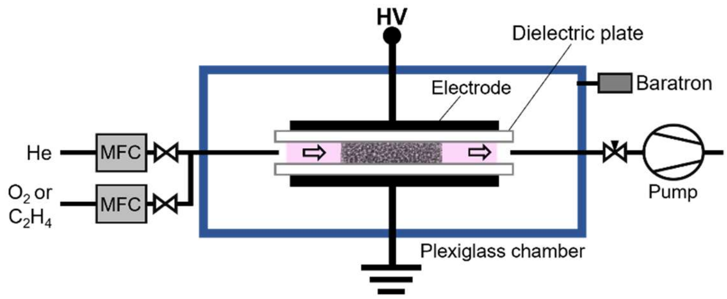

(b)

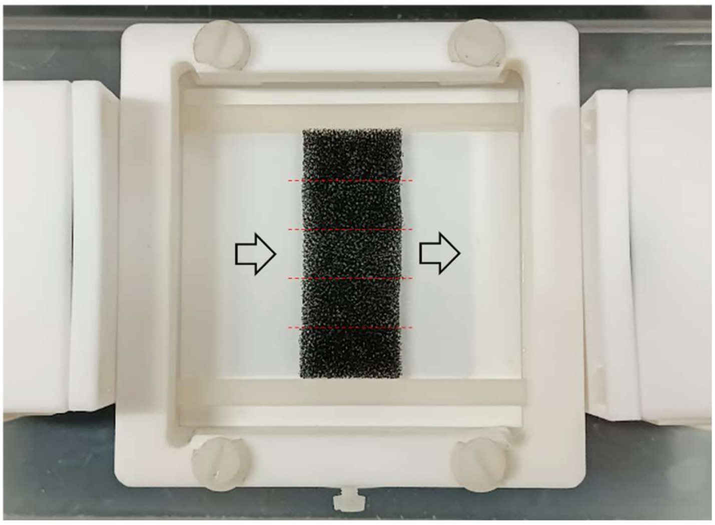

Figure S1. (a) Schematic of the atmospheric pressure DBD reactor; (b) Photograph showing the positioning of five foam samples in the DBD cell (sample length × width × thickness = 20 × 10 × 4 mm^3). The black arrows indicate the gas flow direction.

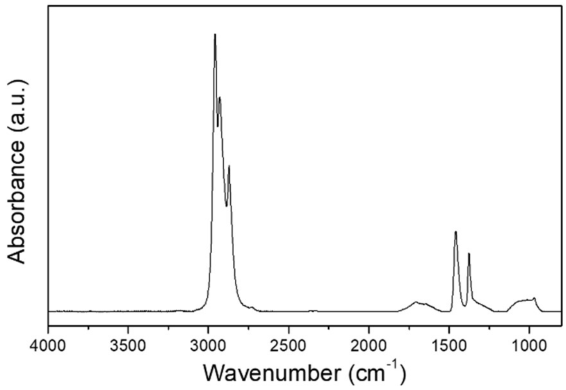

Figure S2. FTIR spectrum of the hydrocarbon polymer thin film deposited on a CaF_2 substrate (Crystran, UK, length × width × thickness = 11 × 11 × 0.5 mm^3) by DBD fed with He/0.5% C_2H_4 mixture.

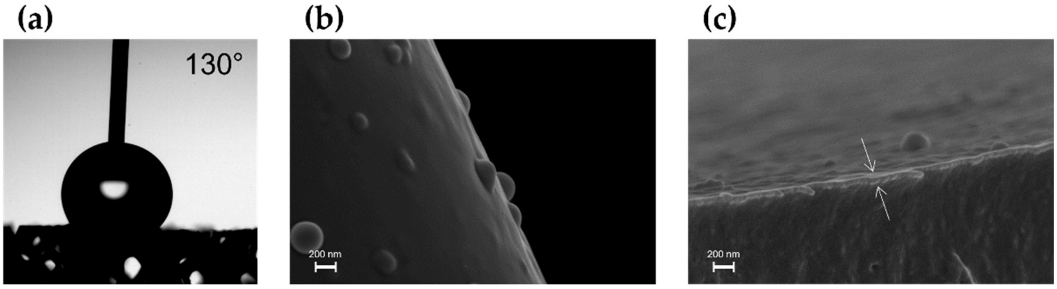

Figure S3. PU foam sample coated with a hydrocarbon polymer thin film deposited by a He/0.5% C_2H_4 fed DBD (deposition time = 10 min): (a) photograph of a water droplet deposited on the foam (WCA = 130 ± 5); (b,c) representative SEM images of the sample interior (i.e., cross-section).

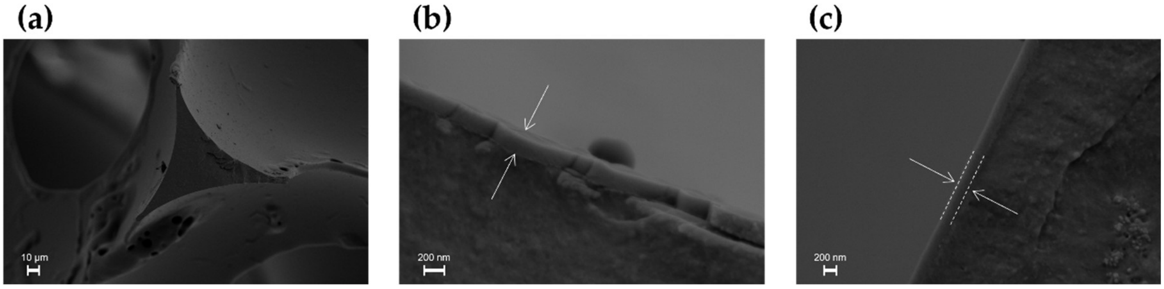

Figure S4. Representative SEM images of a PU foam sample coated with a hydrocarbon polymer thin film deposited for 30 min by a He/0.5% C_2H_4 fed DBD: (a) cross-sectioned ligament; (b,c) different regions of the cross-sectioned ligament where the deposited coating is clearly visible (average thickness of 190 ± 40 nm).

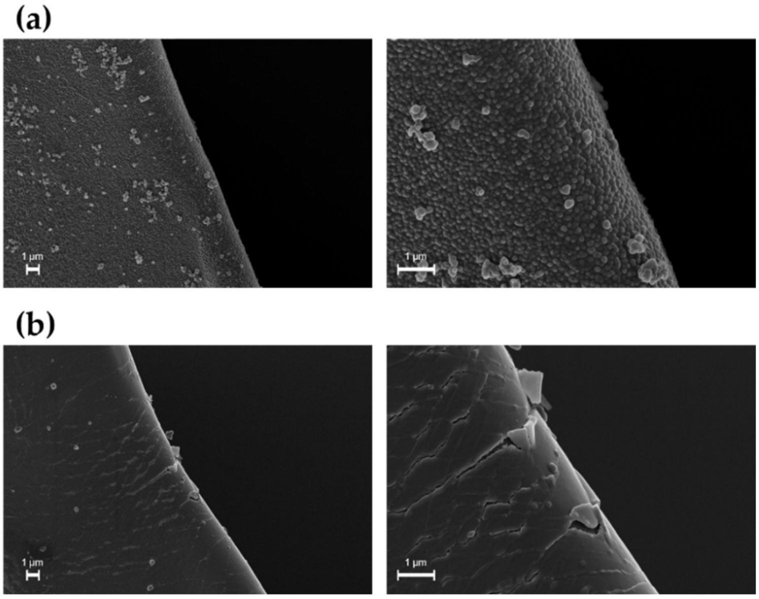

Figure S5. Representative SEM images of plasma-treated PU foam samples after 20 compression-release cycles using a 500 g weight which exerted on them a pressure of 24.5 kPa: (a) PU foam treated with the two-step plasma process (Step 1: He/0.5% O_2 fed DBD, 20 kHz, 1.3 kV_{rms}, 10 min; step 2: He/0.5% C_2H_4 fed DBD, 20 kHz, 1.3 kV_{rms}, 10 min); (b) PU foam coated with a hydrocarbon polymer thin film deposited for 10 min by using a He/0.5% C_2H_4 fed DBD.

Mechanical Characterization

The Young's modulus and the tensile strength of the PU foams were experimentally determined stress-strain curves performing uniaxial tensile tests using an Instron 3365 machine. Tensile testing was conducted at environmentally controlled conditions (20 °C, 40% RH). Dog-bone shaped samples were stretched at a rate of 10 mm/min until failure. Five measurements were conducted for each sample and averaged to obtain a mean value.

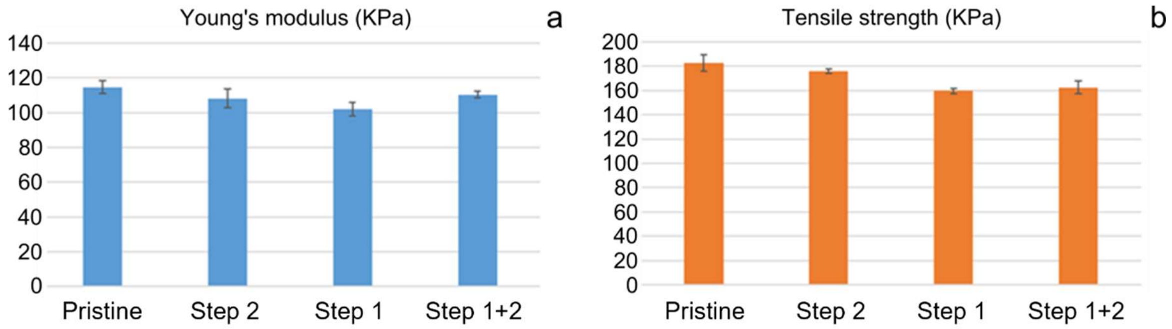

Figure S6. Young's modulus (a) and tensile strength (b) of the pristine and plasma-treated PU foams. Although a slight decrease can be seen, the Young's modulus of the foams is almost unaltered together with the tensile strength, indicating that the materials' modification induced by plasma was not detrimental to the foams mechanical features. Step 1 – etching/nanotexturing: He/0.5% O_2 fed DBD, 20 kHz, 1.3 kV_{rms}, 10 min; step 2 – thin film deposition: He/0.5% C_2H_4 fed DBD, 20 kHz, 1.3 kV_{rms}, 10 min.

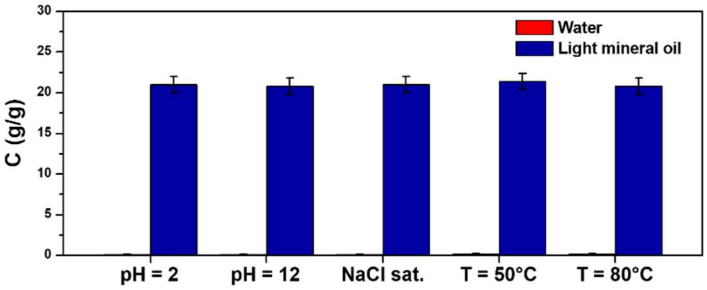

Figure S7. Absorption capacity for water and light mineral oil of the superhydrophobic/superoleophilic PU foam prepared using the two-step plasma process (step 1: He/0.5% O_2 fed DBD, 20 kHz, 1.3 kV_{rms}, 10 min; step 2-: He/0.5% C_2H_4 fed DBD, 20 kHz, 1.3 kV_{rms}, 10 min) after immersion for 60 min in acidic (pH =2) and basic (pH = 10) aqueous solutions, a saturated NaCl aqueous solution and hot water at 50°C and 80 °C.

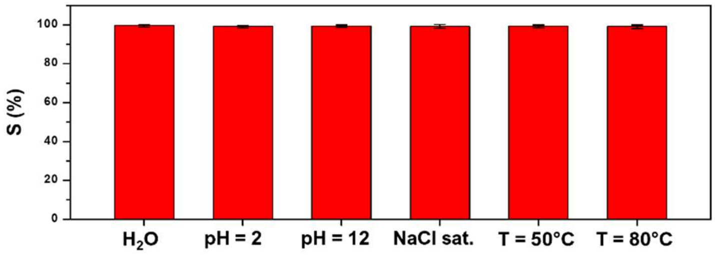

Figure S8. Separation efficiency of the superhydrophobic/superoleophilic PU foam prepared using the two-step plasma process (step 1: He/0.5% O_2 fed DBD, 20 kHz, 1.3 kV_{rms}, 10 min; step 2-: He/0.5% C_2H_4 fed DBD, 20 kHz, 1.3 kV_{rms}, 10 min) for mixtures of light mineral oil with bidistilled water, acidic (pH = 2), basic (pH = 12) and saturated NaCl aqueous solutions as well as for light mineral oil/bidistilled water mixtures heated at 50°C and 80°C.
